# Facilitators and barriers for emergency department clinicians using a rapid chest pain assessment protocol: qualitative interview research

**DOI:** 10.1186/s12913-020-4923-2

**Published:** 2020-01-31

**Authors:** Julia Crilly, Jaimi H. Greenslade, Sara Berndt, Tracey Hawkins, Louise Cullen

**Affiliations:** 1 Department of Emergency Medicine, Gold Coast Health, 1 Hospital Blvd, Southport, QLD 4215 Australia; 2 Menzies Health Institute Queensland, Griffith University, Gold Coast, 4222 QLD Australia; 3 Institute of Health and Biomedical Innovation and School of Public Health and Social Work, Faculty of Health, Queensland University of Technology, 60 Musk Avenue, Kelvin Grove, QLD 4059 Australia; 4 Emergency and Trauma Centre, Royal Brisbane and Women’s Hospital, Butterfield Street, Herston, QLD 4029 Australia; 5 School of Medicine, Faculty of Health and Behavioural Sciences, The University of Queensland, 288 Herston Road, Herston, QLD 4006 Australia

**Keywords:** Emergency department, Qualitative, Chest pain, Acute coronary syndrome, Theoretical domains framework

## Abstract

**Background:**

Guideline-based processes for the assessment of chest pain are lengthy and resource intensive. The IMProved Assessment of Chest Pain Trial (IMPACT) protocol was introduced in one Australian hospital Emergency Department (ED) to more efficiently risk stratify patients. The theoretical domains framework is a useful approach to assist in identifying barriers and facilitators to the implementation of new guidelines in clinical practice. The aim of this study was to understand clinicians’ perceptions of facilitators and barriers to the use of the IMPACT protocol.

**Methods:**

Guided by the theoretical domains framework, semi-structured interviews with nine ED clinical staff (medical and nursing) were undertaken in 2016. Content analysis was conducted independently by two researchers to identify those theoretical domains that facilitated or hindered protocol use.

**Results:**

Domains most often reported as fundamental to the use of the IMPACT protocol included ‘social/professional role and identity’, ‘environmental context and resources’ and ‘social influences’. These factors seemingly influenced professional confidence, with participants noting ‘goals’ that included standardisation of practice, enhanced patient safety, and reduced need for unnecessary testing. The domain ‘environmental context and resources’ also contained the most noted barrier - the need to inform new members of staff regarding protocol use. Opportunities to overcome this barrier included modelling of protocol use by staff at all levels and education – both formal and informal.

**Conclusions:**

A range of domains were identified by ED staff as influencing their chest pain management behaviour. Fundamental to its use were champions/leaders that were trusted and accessible, as well as social influences (other staff within ED and other specialty areas) that enabled and supported protocol use. Research investigating the implementation and perceived use of the protocol at other sites, of varied geographical locations, is warranted.

## Contributions to the literature

- Consistency of practice in managing patients with chest pain in busy emergency departments can be challenging.
- Using the well-recognised theoretical domains framework, facilitators and barriers to the implementation and use of a new chest pain protocol were identified.
- Fundamental to protocol use were trusted and accessible leaders, as well as social influences internal and external to the emergency department.

## Background

Acute coronary syndrome (ACS) is the most common acute presentation of coronary heart disease and the largest single cause of death in Australia [1]. Over 1200 patients present to Australian emergency departments (EDs) every day for investigation of ACS (~ 6% of the 7.8 million ED presentations per year [2]), yet less than 15% will ultimately have the diagnosis confirmed [3]. Historically, risk stratification processes for the identification of patients with ACS required physicians to conduct a detailed clinical assessment incorporating historical features, risk factors, electrocardiography (ECG), and serial troponin testing over at least 6–12 h when using sensitive troponin assays [4–6]. Those with negative results after this initial assessment did not have a diagnosis of acute myocardial infarction but were still at risk for short- and long-term events [7–9]. Therefore, guidelines recommended an objective test in the form of functional or anatomical testing for coronary artery disease [10, 11]. While this approach stratified patients to a near zero short-term risk of ACS, the investigative strategy resulted in a long median length of stay (27.8 h per patient) and incurred high financial costs (median of $2443 per patient) [3].

ED overcrowding has become a global problem [12] meaning that strategies to improve efficiency within EDs are imperative. There also has been a culture shift towards using evidence-based practice to improve patient outcomes. In the area of chest pain assessment, a number of accelerated strategies have been proposed to reduce the time taken to risk stratify patients [13–15]. One such approach was the IMProved Assessment of Chest pain Trial (IMPACT) strategy, trialled in an Australian ED. [16] IMPACT provided new criteria for the risk stratification and management of patients at low- to intermediate-risk for ACS. No change was made for high-risk patients who were risk stratified and managed according to current standard care. Patients *without* high-risk features who were 1) under 40 years of age with 2) no renal failure and 3) no diabetes were considered low risk. Such individuals underwent zero and two-hour ECG and biomarker testing and were discharged with no further testing if results were normal. Intermediate risk patients were those patients with no high-risk features who were 1) over 40 years of age, 2) under 40 years of age with an estimated glomerular filtration rate (eGFR) > 60 mL/min, or 3) under 40 years of age with diabetes. Such individuals underwent 0 and 2 h ECG and biomarker testing followed by inpatient stress testing [16]. Using this protocol, 76% (*n* = 1033) of patients presenting to the ED with chest pain were classified as low or intermediate risk and could undergo accelerated testing [16]. There were no ACS events within 30 days of presentation in the low-risk group and 14 (1.8%) in the intermediate-risk group. The median hospital length of stay was 5.1 h and 7.7 h for the low- and intermediate-risk groups, respectively [16]. When compared against a traditional diagnostic approach [17] of historical controls, for low and intermediate risk patients, hospital length of stay reduced from a median of 24.3 h prior to implementation of the protocol to 7.2 h after implementation of the protocol. Furthermore, the expected costs reduced from $3454 per patient pre-implementation to $2225 post-implementation of the protocol [18]. With the protocol being considered safe, efficient and cost effective, it was implemented as standard care in 2014.

The translation of research into clinical practice can be difficult with evidence showing that it can take around 17 years for innovation to impact patient care [19]. Incorporating protocols into guidelines does not guarantee acceptance; clinicians may not be aware of guidelines, may lack the confidence to act on them, or may not have the knowledge to apply them correctly [20]. Further, findings from tightly controlled clinical trials that only recruit a subset of all patients may not be as successful once implemented within standard care. It may be unwise to invest time and costs to achieve effective translation of a protocol if the benefits seen during the research trial are not as strong when implemented into standard care. Theoretically based frameworks to inform the development, implementation or evaluation of protocols / guidelines and to identify the barriers and enablers to guideline use are therefore helpful when looking to embed research into clinical practice.

The theoretical domains framework has been used to identify barriers and enablers to the implementation of healthcare interventions such as: guidelines for family therapy for families of people with schizophrenia in the community mental health setting [21], guidelines for blood transfusion in the Intensive Care Unit (ICU) setting [22], and safe prescribing of hospital trainee doctors [23]. The use of the theoretical domains framework is relatively new to the ED literature. In the ED setting it has been used for a range of purposes such as to evaluate the use of the Canadian computed tomography (CT) head rule [24], explore factors that influence the use of recommendations for managing mild traumatic brain injury [25], identify barriers and facilitators to the implementation of a screening tool for older people at risk of functional decline and readmission [26], conceptualise and evaluate factors impacting on implementation of a chest pain assessment protocol [27], and guide the development of an intervention to enhance care delivery for people with stroke [28]. The aim of this study was to use the theoretical domains framework to examine clinicians’ use of the IMPACT protocol, whether there were any barriers or enablers to protocol use, and whether there are further opportunities to implement IMPACT.

## Methods

### Design

This qualitative study was guided by a previous retrospective process evaluation undertaken in the ED environment that used the theoretical domains framework [24]. The theoretical domains framework [29] was used to underpin our study as it provides a theoretic approach to ensure that a wide range of theoretical explanations for behaviour are considered. Study write up followed the Standards for the Reporting of Qualitative Research (SRQR) guidelines [30].

### Participants

Participants invited include staff (doctors and nurses) employed on a full-time or part-time basis in the Emergency and Trauma Centre (ETC) at the Royal Brisbane and Women’s Hospital. All staff were informed about the study via an internal email sent from the ETC medical and nursing directors to staff. Within the email, these directors indicated their support of the project and approval of non-clinical time to participate in an interview. A study information sheet was attached to the email and included contact details of the research team so that staff could advise of their willingness to be interviewed. An interview schedule was arranged by a member of the research team. To enable a range of responses, a personal approach was also used to invite other staff. Participants thus comprised a convenience sample. Participants were recruited until data saturation was reached.

All participants were informed that participation in the study was voluntary and would not impact on their employment at the hospital. Individuals were provided information in verbal and written format, offered opportunity to ask any questions, and provided written consent.

### Data collection

Data were collected via one-on-one, face-to-face, semi-structured interviews over a two-week period in 2016. Interviews were conducted by a PhD prepared researcher with previous experience as an ED nurse (JC) in a private room (to maintain privacy and confidentiality and to avoid interruptions) in the ETC. Each interview was voice recorded and transcribed verbatim by an independent assistant. If required, participants were asked to verify aspects of the interview to enhance clarification and interpretation. Transcribed interviews were de-identified.

Semi-structured interview questions were developed to enable an understanding of behaviour change [29] regarding their assessment of acute coronary syndrome, and in particular the IMPACT protocol. Interview questions were adapted from those used in past research [31] (see Additional file 1). Questions were piloted with one senior doctor and a nurse to assess clarity. Examples of questions included: What is your understanding of the recommendations regarding accelerated chest pain assessment? Where and how did you learn about the pathway? To what extent do you think the chest pain pathway is being implemented? Can you give me a recent example of it happening? What problems have you encountered? What would happen if you didn’t use the pathway? Would you say the benefits outweigh the costs? Do you think that this pathway could be implemented in other hospitals?

### Rigor

Rigor was maintained through the use of recommended strategies [32–34]. An external interviewer (not employed at the hospital) performed the interviews to minimise the risk of bias and threat of potential interviewee – interviewer clinical professional relationship concerns. To enhance credibility, different multi-disciplinary perspectives were purposively sought and participant quotes from various sources are presented. Other researchers were involved with the analysis to reflect dependability. Participant characteristics and contextual data are reported so that transferability can be considered by readers.

### Data analysis

Interview data were analysed using the theoretical domains framework [29]. Within this framework, there are 14 domains that explain behaviour change. These include 1) knowledge; 2) skills; 3) social/professional role and identity; 4) beliefs about capabilities; 5) optimism; 6) beliefs about consequences; 7) reinforcement; 8) intentions; 9) goals; 10) memory, attention and decision processes; 11) environmental context and resources; 12) social influences; 13) emotion; and 14) behavioural regulation. The interview questions aimed to identify information relevant to these domains. Content analysis was conducted independently by two researchers (SB and JC) to identify theoretical domains and constructs as well as to identify which domains were barriers and or enablers to change. It also involved identifying patterns across data sets (e.g. frequency of noted constructs within each domain in each and all interviews). The domains and constructs coded by each researcher were compared and discussed with consensus reached using a third researcher (JG) where required. Thematic analysis was performed in six phases including familiarization with data, generating initial codes, searching for themes among codes, reviewing themes, defining and naming themes, and producing the final report [35].

## Results

A total of nine participants were interviewed. These participants represented both medical (*n* = 4) and nursing (*n* = 5) staff working at the ETC where the protocol was implemented. Three participants were male and the average age of participants was 40 years (SD: 10.3). The average years of experience in their profession was 17 (SD: 9.2), with 12.6 years (SD: 6.6) working in the ED. Interviews lasted on average 32 min (range: 22 to 44 min).

Of the 14 domains, 8 were identified as relevant to the context of chest pain assessment and management and the use of the IMPACT protocol (see Table 1); 5 domains clearly reflected enablers to protocol use. There was overlap in three domains (memory, attention and decision and processes; skills; and environmental context and resources) where both barriers and enablers were evident. The key themes underpinning these domains, along with examples of quotes are presented in Table 1.

**Table 1:** Theoretical domains, themes and example participant comments

Theoretical Domain	Theme	Example Participant Comments
Knowledge	Knowledge about the protocol	“Everyone uses it. Everyone knows about it.” (P149)
	Knowledge of the contents of the protocol	“We still have the low-risk, intermediate-risk, and high-risk groups, but our approach to investigations within those groups has changed with the new process. So specifically, for the low-risk group, so patients less than 40, they now only need to have two troponins and two ECGs two hours apart, and then they are able to be discharged home if they are both negative or normal. Whereas previously they would need a 6-h troponin (Tn) or stress test, potentially. The intermediate risk group, likewise have troponin at two hours and two ECGs and if those are negative they can then have a stress test. So shortening the serial assessment from 6 h down to 2 h.” (P150)
Social/Professional Role and Identity	All professionals have a role within the pathway	“We have to be trained to like to be able to take bloods but once you are able to do that, we can initiate troponin etc. if we believe that they meet the criteria for the chest pain pathway. That’s not frowned upon, that’s encouraged by doctors. Chest x-rays, they have to be signed off by a medical officer, but we can go to them and say “the patient requires a x-ray for the chest pathway” and they will sign off” (P152) “From a nursing perspective we’re aware of it, but ultimately the responsibility of going on whatever sort of, you know, low-, intermediate-, or high-risk is up to the medical staff and they oversee medical staff who are overseeing that” (P151)
	The protocol fostered teamwork and cooperation	“It needs to be a team effort” (P149) “I put them on high risk and then have a chat with cardiology and then they’re admitted” (P154) “I think most people are on board with it, in terms of our medical colleagues in cardiology as well” (P154)
	The protocol provides professional confidence	“I think it tends to protect the practitioner … because I’m actually following what is actually agreed upon between the departments” (P148) “But it’s very reassuring to be able to do that second troponin and it very rarely delays their stay. So, I think probably people are still getting put on the pathway because it is considered so low-harm and low-cost. And it’s reassuring” (P150)
	There is a trusted leader supporting the protocol	“Here, we believe in [study lead], it’s easy if I say [study lead] said this is what we’re do, this is what we do.” (P148)
Beliefs about Capabilities	The protocol is easy to use	“It’s easy to use and it cuts out any indecision” (P149)
	Confidence in using the pathway	“If my seniors and consultants and registrar are using the pathway then I feel empowered, I guess, and confident that they have put their confidence in the pathway so that I should be able to as well” (P154) “I wouldn’t make that part of my triage because I don’t have confidence in my entire grasp of it. And also, I think it needs to be something led from a team perspective” (P149)
	Empowerment	“… created autonomy as nurses because since the pathway has been brought out, if we think they are a very low-risk pathway then we won’t initiate blood work on them. But if they meet the intermediate- or high-risk pathway then we, as nurses, can initiate the measurement of the troponin and the cut off markers, so,. I think it’s given us, if anything, a little bit more autonomy” (P152)
Goals	Standardized evidence- based practice	“They are a pathway and a guide so there are times when patients don’t fit the mould and it’s important that we don’t try to make them fit the pathway. But for ensuring consistency of practice and making sure that we are working with the best evidence I think I all pathways can be very helpful” (P150) “it’s like a checklist thing to make sure that you are achieving what the researchers have proven to be the best for the patient” (P151)
	Improved patient flow, reduced length of stay and reduced cost	“Faster time to assessment; faster time to discharge; less length of stay; saving money for the health services. We, like I said earlier, don’t tend to over investigate them as much anymore.” (P152) “It’s good for the patient and its good for patient flow” (P 148) “Total length of stay was much shorter than it otherwise would have been” (P150) “We’re picking up things a lot quicker now” (P154) “We know the destination for the patient, we can pre-plan our movement, bed bookings, booking different types of investigations, all the self-initiated investigation of the patient” (P152)
	A goal is to enhance patient safety	“They’re [pathway] a great safety net” (P 147) “It’s the outcomes that have been achieved from it have proved positive. And you know we are working collectively towards again, improving patient outcomes” (P151) “it’s just a, a way that we can more efficiently and better treat all our chest pain pathway patients. And we manage them and, you know, allocate appropriate resources and time and make it safe for the patient” (P149)
	Reduced unnecessary testing	“It avoids unnecessary inpatient testing” (P154) “they’re not getting stress testing and they’re not getting those sorts of things” (P154)
Social influences	The use of the pathway is a group norm	“I would see it as an exception if people don’t use it. Like I would say the majority of people use it” (P153) “In Emergency, I’m not aware of any of us who are using the previous process. All of us would be, well I believe we are all using it” (P150) “Basically, everyone refers to it” (P148) “it’s sort of embedded in the culture” (P151)
	There is social pressure to conform to the group norms	“They hear the “pathway.” Because the nurse prompts “are they going to, will we go and put them on the pathway, because we are taking the blood. Do you want a troponin; okay put them on the pathway”. They are sort of going “Oh what’s a pathway?” And then they’ll come along and just it’s just like they’ve come along for the ride with you.” (P147) “They’ll go work elsewhere and what they do elsewhere is slightly different. And when they initially start here they’ll probably actually just do what they are familiar with. But once it’s been pointed out “hey this is what we do here” and then I think most of them tend to follow it” (P148)
	Intergroup support facilitates protocol use	“There’s actually agreement between us and inpatient teams, so there’s actually no disagreement to what needs to be done” (P148) “I think it must be easier for the inpatient units to accept referrals from us, having a protocol like this to drive their admissions they know that it’s there, they’ve had buy in to the process so that allows them to monitor” (P149) “In ED it’s very well regarded. Nursing staff like it, again because they know what’s going to happen with a patient. Hopefully there is less individual practitioner variability. That’s a good thing. The medical staff like it, again for the same reasons. It’s clear what the processes are and it won’t depend on who the consultant supervising is as to what will happen with a patient. Inpatient teams, may have been sceptical at first but I think they quite like it also because it’s probably diverted a lot of referrals that might have come their way” (P150)
Memory, Attention and Decision Processes	Staff reported that they were not always able to remember the guideline.	“You don’t need to keep looking at the poster once you know the principles, you know the risk factors, you know what it is, it’s easy to remember and the fact that it’s colour coded” (P147) “I don’t know them off by heart” (P149) “You’ll see people looking at it all the time just to confirm that patients really are low risk or intermediate risk. ‘Cause they would previously have fallen into different group. And you’ll see nurses looking at them, you’ll see the residents and the registrars as well. And you’ll see consultants checking, especially the diabetic patients, just making sure that we are doing the right thing. And like, I think that’s great. I don’t think there’s any problem with people checking it. It’s important that we are doing the right thing” (P150) “I can’t tell you it verbatim, but I know it’s there.” (P151)
	Decision making was aided through the use of a standardised protocol	“There’s some really clear, clear guidelines and they’re quite easy to follow and really spells it out. And everyone’s kind of on board with it as well, so, bit of a no brainer. But very logical and clear guidelines so, and just helps you to sort of come up with your decision making as well” (P154) “It just helps you, yeah, not make mistakes based on the evidence from the research. And you know if you’re busy, it’s always busy, and chaotic in an emergency department, so it gives you again the structured approach so you’ve got some steps to follow, you know what you are doing” (P151)
Skill	Not all individuals have the skills required to identifying patients eligible for the pathway	“It’s an easy route for less experienced decision makers to confuse. So more junior registrars for example, especially on nights, it can, you can find patients who have been put on this pathway but who it’s not appropriate” (P150) “One of the things that people still struggle with is that is it a PE or is it a, is it cardiac. And so they get put on a double protocol. They get a PE protocol and a chest pain pathway. You know, like it’s almost, at times it’s almost without, without thinking and you say, well we do this, we’ll follow this. But sometimes we don’t really think enough” (P148) “Usually women who have come in with atypical pain which can sometimes be difficult to differentiate. And they will, or should have, a PE exclusion first. Occasionally that does happen, and we just need to keep that in front of our mind” (P150)
	There is some de-skilling	“I think it takes away from clinical, your own individual clinical assessment. But that’s happening with all pathways. Clinical deskilling, definitely. Definitely. Especially with the junior medical staff. They hear the “pathway.” Because the nurse prompts “are they going to, will we go and put them on the pathway, because we are taking the blood. Do you want a troponin; okay put them on the pathway” … (P147)
	Improving skills through interpersonal communication	“As a nurse, like for me, if I, when I look after the chest pain patient, I think in my head where they fall. I go to the pathway to double check, talk to the doctors and say is this right?” (P149) “When I call them [cardiology] on that I actually say, this person’s on the high-risk chest pain pathway. I just, I like, some of the features that are in this high-risk box really. And it’s fairly straight forward and self-explanatory and usually cardiologists and all the registrars that we talk to are very receptive and they’re pretty good” (P154)
	Staff turnover and training new staff is a barrier to protocol use	“Staff are changing so often and it’s really hard to keep up with the training or the education for this so it slows down the staffing abilities to follow the protocol accurately” (P152) “We’ve had a big change over staff have, it probably would be a good idea in some way for new grads and all that sort of staff to be aware of what this is cause they have so many other things that they need to achieve that they probably don’t, they’re not always aware of this sort of thing” (P151) “So the registrars change about every 6 months usually. So, the first week of their term under, they get the hospital orientation and then a department orientation and then I think they get on the Thursday, they have a more in-depth orientation about clinical and education and training issues” (P150)
	Having an accessible, clear, easy to follow protocol is a facilitator	“It’s behind the computer when you walk up the stairs from resus, it’s behind the wall on a computer there and it’s blown up, really. It’s great. But it’s like, it’s blown up really big. It’s a spot that’s easy to access, it’s close to resus side where most of the time we use it. You can get it off the [ED resource website] as well. There’s copies of it in each resus bay” (P149) “The chest pain pathway is very accessible. Like it’s something that we use very frequently, everyone knows a little bit about it, and even if you don’t know a lot about it you know where you can go and stand at the wall. And it’s one of those algorithms, like the actual, it’s easy to read, it’s easy to follow” (P149) “From a visual perspective, it’s not busy. There’s not a lot of wording in it. It’s colour coded. It’s got arrows showing flow” (P149) “We’ve got posters around the department just to prompt us if we’re uncertain which is handy” (P150)
Environmental Context and Resources	Person x person interaction overcomes the barrier of training new staff	“From my encounters it’s usually when they are on the floor so they’ll encounter this patient and there’s always a senior staff member somewhere and we will inform them of this pathway and I’d have to check with our educator to see if there is a formal education process surrounding it. It seems to be floor and word of mouth” (P152)
	The resources required to utilise the protocol are provided by the department	“We’ve got 24-h access to troponin here. But I suppose there would be places that might have bedside troponin point of care testing, which would be okay. We’ve got very good access to stress tests. Quite often same day, but if not same day, then next day, even on the weekend. And I think that has been crucial and it’s shown that this pathway has been as successful as it has been” (P150) “We have the ability to do the blood tests along with the specialised accelerated blood tests with the delta Z times et cetera. We have the ability to access ECGs easily, along with chest x-rays, we also have the capacity to do exercise stress testing (EST), 7 days a week. It’s for less resourced centres, this may not be appropriate” (P152)

### Enablers to protocol use

Domains that reflected enabling facets of the implementation of the IMPACT protocol included i) knowledge, ii) social professional role and identity, iii) beliefs about capabilities, iv) goals, and v) social influences.

#### Knowledge

All participants were aware of the IMPACT protocol and used it daily when working clinically. Participants were aware of the branches within the pathway (i.e. high risk, moderate risk, low risk), and most knew the criteria for each. Aspects noted to underpin protocol use included: education provided regarding the protocol; continued and visible access to the protocol (enlarged, easy to follow, colour print-out located in areas where a patient likely to require protocol use are located); and that it was driven by a respected and trusted leader within the field located in their department.

#### Social professional role and identity

Despite the variation in interview participants (nurses and doctors, junior and senior staff), all noted that they had a role to play regarding IMPACT – be it assessment, management or referral. Participants were quite clear in terms of their professional boundaries but noted that IMPACT fostered a sense of teamwork and cooperation. The reduced variation in practice that IMPACT offered was welcomed by staff in the ED and the protocol provided professional confidence that assisted with decision-making and collaboration with inpatient specialists. One of the key facilitators of the protocol was that a trusted professional colleague led it and this increased their motivation to use the protocol.

#### Beliefs about capabilities

Participants reported that the protocol was easy to use and empowered them to make decisions. They felt encouraged to initiate the pathway if they felt that the patient was suitable. The IMPACT protocol provided some practitioners with a sense of confidence in managing patients with chest pain. However, one respondent reported that they did not have the confidence to use it except within the context of the treating team. One also noted that relying on a protocol might result in less use of clinical judgment and higher risk of missing a diagnosis.

#### Goals

Goals of the IMPACT protocol implementation were noted by participants to include: standardisation of evidence-based practice; improved patient flow, reduced cost and reduced length of stay; enhanced patient outcomes; and the reduced need for unnecessary testing.

#### Social influences

The use of IMPACT was normative, with respondents reporting that everyone uses it. Group conformity and social pressure were noted factors to enhance protocol use. Intergroup support from inpatient teams was also highlighted as a facilitator to the protocol. The standardisation of practice that the protocol provided also helped with inter-professional communication.

### Cross over

Three domains contained both barriers and facilitators of protocol use. These included memory, attention and decision and processes; skills; and environmental context and resources.

#### Memory, attention and decision processes

There was a strong sense of knowledge of the IMPACT protocol and most participants generally knew the criteria. However, participants reported that they could not always remember the guideline content. They noted that posters placed throughout the department and access to the guideline on the ED electronic repository assisted them when needing to clarify or recall criteria to inform their decision-making. They also welcomed the opportunity for standardisation of practice that assisted decision-making in an area that was previously unclear.

#### Skills

Respondents noted that it required specific skills to identify which patients are eligible to be placed on the pathway. This was a barrier for less experienced clinicians who may have difficulty identifying appropriate patients for inclusion. De-skilling was also identified as a concern as the use of a pathway reduced the requirement for clinical decision-making. Skill building through positive interpersonal encounters was identified as a positive aspect of the pathway.

#### Environmental context and resources

This domain contained the most noted barrier to protocol use, that is, the need to inform new members of staff. However, there were also facilitators identified to overcome this barrier. These facilitators included the accessibility to a clear, easy to follow protocol that was supported by the organisation. The person-person interaction involved modelling of protocol use by staff at all levels and education – both formal and informal. Another facilitator identified by participants was that resources needed to follow the protocol were readily available within the ED.

## Discussion

The assessment of chest pain comprised considerable research focus, with a number of accelerated pathways having been developed [13, 14, 16]. However, few pathways have been implemented as standard care. The IMPACT pathway has been implemented following positive patient and service outcomes in terms of safety and efficacy [16]. The current study highlights facilitators and barriers to the implementation of this protocol.

This study supports previous ED research [24, 25, 27, 28] in finding that the theoretical domains framework provides a useful approach to retrospectively identify barriers and enablers to protocol use in the ED. It also supports the considerable literature utilising this framework to explain behaviour change across a variety of interventions (e.g. back pain management, blood transfusion practices, prescribing errors, family intervention for people with schizophrenia), population groups (e.g. doctors – General Practitioners, ICU physicians, trainee doctors, mental health team members – social workers, nurses, team managers, psychologists, psychiatrists), and settings (ranging from primary care, hospital setting, ICU, community and mental health) [21–23, 36].

Domains that reflected enabling facets of the implementation of the IMPACT protocol included knowledge, social professional role and identity, beliefs about capabilities, goals, and social influences. Staff reported that they were knowledgeable in the pathway, that they were confident in implementing the protocol, that it strengthened relationships within the department and across departments, and that its use had become normative within the department. These domains have been noted in other research regarding protocol use in the ED. Knowledge and social/professional role and identity have been recognized as key to the successful implementation of a previous chest pain pathway [27] and being confident in applying a clinical decision rule and social influences have been identified as facilitators to using a CT rule [24]. However, Curran et al. [24], noted that while knowledge about the CT rule was high, this was not a key driver to implementation, with various contextual factors outweighing knowledge.

Three domains incorporated both facilitators and barriers in the current study. Cross over identified within a domain is not uncommon. Curran et al. [24], reported that beliefs about the consequences of the Canadian CT head rule were both facilitators (supports decision making and reduces radiation exposure) and barriers (hinders patient flow in the department). Similarly, Craig et al. [28], conducted a review of interventions to manage acute stroke in the ED and identified multiple studies where the environment, context and resources domain and skills domains incorporated both facilitators and barriers. In our study, with regard to memory, being able to remember and act on a new pathway was identified as a barrier. Resources were also identified as a concern; with staff feeling they did not have the resources to train new staff to implement the pathway. These domains (as barriers) have previously been identified in the ED setting [24]. However, it was noted in the current study that the provision of visible and clear information for staff on the protocol were facilitators that alleviated these barriers. Previous research using the theoretical domains framework has noted a number of additional barriers to implementing new pathways or guidelines in the ED that were not identified in the current research. For example, beliefs about consequences, in particular, beliefs about increased time requirements on an already heavy workload, have been identified as barriers [24, 26]. Lack of skills or confidence around implementing new protocols has also been identified as a concern [24, 25]. These barriers may not have emerged in the present study as the IMPACT pathway is a simple accelerated protocol that saves time within the ED. Staff had also undergone extensive training in the pathway from the clinical lead and from research nurses who were involved in trialling the protocol.

Implementation depends on clinicians and managers changing a variety of behaviours [21]. If interventions are to be successful, they need to be grounded in an understanding as to why health professionals do or do not change their behaviour [21]. Historically, studies using the theoretical domains framework tend to focus (understandably) on the barriers, rather than the enablers to practice change. In this study, enabling factors underpinning why health care professionals changed their behaviour included: having knowledge of the protocol, being part of a team that embraced the process, knowing and trusting the change leader, having an awareness that the protocol implemented was evidence based and the ultimate belief that it was what was best for the patient, the ED, and the health system. These are factors for consideration if implementing the IMPACT or other protocols elsewhere.

### Limitations

Limitations to this study include the small sample size, however data saturation was reached, and the sample reflected a depth of clinician types (doctors and nurses) and experience (junior and senior). This study was undertaken at one site. Whilst findings may not be generalizable to other EDs where different processes are used for chest pain management, they may be helpful for EDs that intend to implement the IMPACT protocol. Finally, the theoretical domains framework is typically used prior to implementing an intervention designed to improve practice. We used the theoretical domains framework following the implementation of a protocol designed to standardise practice. However, using the protocol for this purpose was useful in identifying where there is further opportunity for improvement, and identifying which domains facilitated implementation and protocol use.

## Conclusions

Findings from this study indicate that staff were knowledgeable about the IMPACT protocol and used it frequently. Whilst staff may not have an in-depth knowledge of requirements of the protocol, the ready access to a variety of resources and education enabled its use. Although there was uncertainty regarding the cost-benefits, there was agreement that the standardisation in practice the IMPACT protocol offered was of benefit to the patient, the practitioner and the health service. Imperative to the development and implementation of the protocol was the driver (an experienced staff specialist with an academic background in chest pain) who was seen as a leader, had rapport, and trust of staff at all levels within the ED. Engagement from leaders from other in-patient areas (e.g. cardiology, medicine) was seen as imperative to protocol establishment and subsequent use. Findings from this study can be used to support the implementation of the protocol at other sites as well as further refine and improve the way that the IMPACT protocol is translated into clinical care in the future.

## Supplementary information

**Additional file 1.** List of questions used to guide interviews

## Data Availability

The dataset is not publicly available due to confidentiality policies.

## Abbreviations

ACS: Acute coronary syndrome
ECG: Electrocardiogram
ED: Emergency Department
eGFR: estimated glomerular filtration rate
ETC: Emergency and Trauma Centre
IMPACT: Improved Assessment of Chest Pain Trial
