# Supplementary material for: Facilitators and barriers for emergency department clinicians using a rapid chest pain assessment protocol: qualitative interview research
Source: BMC Health Serv Res. 2020 Jan 31;20:74. doi: 10.1186/s12913-020-4923-2 (PMC6995126; doi:10.1186/s12913-020-4923-2)
Supplement: Supplementary file 1 — Additional file 1. List of questions used to guide interviews [file 12913_2020_4923_MOESM1_ESM.docx]

**Additional File 1**

Facilitators and barriers for emergency department clinicians using a rapid chest pain assessment protocol: qualitative interview research

**List of questions used to guide interviews***

[*Notes in these parentheses are for the interviewer*]

1. Have you heard about the revised Acute Coronary Syndrome protocol produced by Louise and colleagues?

If yes – Are you aware of the recommendations regarding accelerated chest pain assessment?

If yes – What is your understanding of the recommendations regarding accelerated chest pain assessment?

If yes - Where and how did you learn about the pathway?

If no – The recommendations are… [*show pathway*]

1. To what extent do you think the chest pain pathway is being implemented? Can you give me a recent example of it happening? Do you know how to provide accelerated chest pain assessment? Do you think that other members of your team know how to provide accelerated chest pain assessment?
2. What are your views about pathways in general? Does that opinion apply to this pathway? Do you think it is an appropriate part of your job to be following the recommendations in the pathway? Would following the recommendations in the pathway create a problem for your professional autonomy?
3. Is the pathway easy or difficult to use? What problems have you encountered? What would help you to overcome these problems?
4. What are the consequences of using the accelerated chest pain protocol (prompt: advantages and disadvantages for the patient, you as the practitioner, the health service)? Would you say that the benefits outweigh the costs? What would happen if you didn’t use the pathway?
5. Do you feel that you should be using the accelerated chest pain pathway? Are you motivated to use the accelerated chest pain pathway? Does using the accelerated chest pain pathway conflict with any of your other goals as a health professional?
6. How often do you use the accelerated chest pain pathway? What are your reasons for not using the accelerated chest pain pathway (prompt: for attention, forgetting, time constraints, etc.)
7. To what extent do resources influence whether you use the accelerated chest pain protocol (prompt: for existence of trained staff, time constraints, etc.)?
8. What do you think the views of the other team members are? Do these views influence whether you use the accelerated chest pain protocol?
9. Do you think that any emotional factors influence whether the accelerated chest pain protocol is used? And what about for you?
10. Are there procedures or ways of working that encourage the use of the accelerated chest pain pathway? If you see a patient and decide they should have the accelerated chest pain pathway, what are your next steps?
11. Do you think that this pathway will be a sustainable change? (If yes – why – if no – why not?) Do you think that this pathway could be implemented in other hospitals? In the future do you see any changes to how chest pain assessment or management occurs? How confident would you be that those changes wold be supported?

*Questions adapted from Miche et al., 2007 (31)
